# Behavior change pathways by which digital wearable devices support exercise self-management in type 2 diabetes: a scoping review with machine learning–assisted text mining

**DOI:** 10.3389/fpubh.2026.1871682

**Published:** 2026-07-30

**Authors:** Wei Sun, Dong Xie, Wenhui Hou, Menglin Zhang, Chendi Wang, Ziru Wang, He Yang, Guodong Xu, Haochen Dai

**Affiliations:** 1 School of Nursing, Changchun University of Chinese Medicine, Jilin, China; 2 School of Public Health, Jilin University, Jilin, China

**Keywords:** digital wearable devices, exercise self-management, machine learning, text mining, type 2 diabetes mellitus

## Abstract

**Background:**

Long-term self-management is essential for patients with type 2 diabetes mellitus, yet exercise management remains one of the weakest components of self-care. With the advancement of digital health technologies, digital wearable devices have increasingly been used to support diabetes management. However, the pathways by which these devices facilitate exercise behavior change remain insufficiently understood.

**Objective:**

This scoping review aimed to systematically map the existing evidence on digital wearable devices supporting exercise self-management in patients with type 2 diabetes mellitus, identify their behavior change pathways, and use machine learning–assisted text mining to examine major research themes and hotspots, thereby informing the development of targeted interventions.

**Methods:**

A scoping review was conducted across PubMed, Scopus, Web of Science, Embase, CINAHL, PsycINFO, and the Cochrane Library, covering studies published from database inception to March 2026. The search strategy was constructed using terms related to type 2 diabetes mellitus, digital wearable devices, exercise self-management, physical activity, and behavior change. Machine learning–assisted literature mining was used to identify thematic patterns in the included studies. The study selection process and overall workflow were conducted in accordance with the PRISMA Extension for Scoping Reviews (PRISMA-ScR).

**Results:**

11 studies were included in the review. The evidence indicates that digital wearable devices facilitate exercise self-management in patients with type 2 diabetes mellitus primarily through self-monitoring, real-time feedback, goal setting, motivational activation, social support, and enhanced self-efficacy. Machine learning–assisted text mining further showed that the literature in this field is mainly centered on exercise intervention design, behavioral regulation mechanisms, glycemic monitoring outcomes, and physical activity tracking.

**Conclusion:**

Digital wearable devices appear to support exercise self-management in people with type 2 diabetes through multiple, interacting behavior-support pathways rather than through any single function alone. This review identifies recurring patterns in the literature rather than making causal inferences, and may inform the development of personalized digital interventions for exercise self-management based on behavior change theory.

**Systematic review registration:**

https://doi.org/10.17605/OSF.IO/PBG8W.

## 1 Introduction

Type 2 diabetes mellitus (T2DM) is a chronic metabolic disease characterized by insulin resistance and inadequate insulin secretion. According to the International Diabetes Federation, approximately 589 million adults worldwide were living with diabetes in 2024, and this number is projected to rise to 853 million by 2050 (1), making diabetes a major and growing public health challenge. Owing to its long disease course and high risk of complications, patients with T2DM require not only long-term pharmacological treatment but also sustained self-management, including dietary control, blood glucose monitoring, and regular physical activity (2). Among these components, exercise is a cornerstone of comprehensive T2DM management, with well-established benefits for improving insulin sensitivity, promoting glucose utilization, controlling body weight, and reducing the risk of complications (3). However, compared with dietary adherence, medication taking, and blood glucose monitoring, exercise self-management is often one of the most difficult self-care behaviors for patients with T2DM to initiate and maintain over time (4). With the rapid advancement of digital health technologies, digital wearable devices have increasingly emerged as important tools for chronic disease management. These devices, including smartwatches, fitness bands, and continuous glucose monitoring (CGM) systems, offer functions such as real-time monitoring, data recording, and feedback reminders. By objectively capturing exercise-related indicators, they may enhance patients' awareness of their own behaviors and support their engagement in physical activity and behavioral adjustment (5, 6). Although previous studies have demonstrated the potential benefits of such devices, most have focused primarily on outcome measures, with limited attention paid to the pathways through which these technologies facilitate exercise self-management. From a theoretical perspective, behavior change theories provide an important framework for understanding the initiation, maintenance, and internalization of health behaviors in people with chronic conditions (7). Current interventions targeting exercise behaviors in T2DM are commonly informed by theories such as social cognitive theory (SCT), self-determination theory (SDT), the transtheoretical model (TTM), and goal-setting theory (8, 9). These theories explain health behavior change from different but complementary perspectives, including self-efficacy, autonomous motivation, stage-based behavioral transition, and goal reinforcement. Notably, the core functions of digital wearable devices—such as self-monitoring, real-time feedback, prompts and reminders, and goal management—closely align with key constructs embedded in these theories. This suggests that such devices may influence exercise self-management in T2DM through pathways including enhanced self-efficacy, motivational activation, improved self-regulation, and support for sustained behavior (10, 11). Nevertheless, evidence synthesis remains lacking on the behavior change pathways through which digital wearable devices support exercise self-management in people with T2DM from a theory-informed perspective. Therefore, this scoping review aims to systematically map the existing research on digital wearable devices supporting exercise self-management in T2DM, with a particular focus on identifying the behavior change pathways involved in the initiation, maintenance, and optimization of exercise behaviors. In addition, machine learning–assisted text mining will be applied to analyze thematic distributions, research hotspots, and the emerging knowledge structure of this field. By doing so, this review seeks to clarify the current state and developmental trends of the literature and to inform the future design of theory-driven digital interventions for exercise self-management.

## 2 Methods

### 2.1 Study design

This study was conducted as a scoping review, with its design and reporting guided by the PRISMA Extension for Scoping Reviews (PRISMA-ScR) (12). The review protocol was registered and made publicly available on the Open Science Framework (https://doi.org/10.17605/OSF.IO/PBG8W). In line with scoping review methodology, this review aimed to map the breadth of the existing evidence and clarify the conceptual structure of the field, rather than to quantitatively synthesize effect sizes or appraise the methodological quality of individual studies.

### 2.2 Research question

To clarify the research question and define the scope of the review, a preliminary exploratory search was first conducted to identify the literature on digital wearable devices supporting exercise self-management in patients with T2DM. The primary aim of this scoping review was to systematically map and synthesize the existing evidence on the use of digital wearable devices to support exercise self-management among individuals with T2DM, with particular attention to the potential behavior change pathways involved. Based on the findings of the preliminary search, the review was guided by the following research questions: (1) What are the main application contexts and research focuses of digital wearable devices used to support exercise self-management in patients with T2DM? (2) What key behavior change pathways have been described through which digital wearable devices may facilitate the initiation, maintenance, and optimization of exercise behaviors? (3) What thematic patterns and conceptual relationships have been identified among device functions, behavior change processes, and exercise self-management outcomes in the existing literature? Accordingly, the overarching research question of this review was: What behavior change pathways are involved in the use of digital wearable devices to support exercise self-management in patients with T2DM, and what are the current research trends and key discussion points in this field?

### 2.3 Search strategy

In accordance with the PRISMA Extension for Scoping Reviews (PRISMA-ScR) guidelines and the Arksey and O'Malley framework (12, 13), a comprehensive literature search was conducted to identify studies examining behavior change related to the use of digital wearable devices to support exercise self-management in patients with T2DM. The following electronic databases were searched: PubMed, Scopus, Web of Science, Embase, CINAHL, PsycINFO, and the Cochrane Library. The search covered all records from database inception to March 13, 2026. A combination of controlled vocabulary terms and free-text keywords was used, including terms related to digital wearable devices, behavior change, exercise self-management, physical activity, and type 2 diabetes mellitus. Boolean operators (AND, OR), truncation symbols (^*^), and field tags (e.g., [Title/Abstract]) were applied as appropriate to ensure a comprehensive search. The PubMed search strategy served as the initial framework and was translated and adapted individually for the other databases to account for differences in controlled vocabulary, such as MeSH and Emtree, field tags, truncation rules, and proximity operators, while retaining the same core search concepts. The complete PubMed search syntax is presented in Supplementary Material S1 as a representative example. Searches were restricted to publications in English or Chinese because these were the languages that the review team could assess reliably; this restriction may have introduced language-related selection bias. In addition, the reference lists of the included studies and relevant reviews were manually screened to identify any additional eligible studies.

### 2.4 Eligibility criteria

#### 2.4.1 Inclusion criteria

Studies were included if they met the following criteria: (1) the study population comprised adults aged 18 years or older with T2DM; (2) the study explicitly examined the impact of digital wearable devices on exercise self-management behaviors in patients with T2DM, or addressed behavior change pathways, behavior change techniques, or mechanisms related to such interventions; (3) the study was an original empirical study, including cross-sectional, cohort, interventional, or mixed-methods research; and (4) the publication was written in either Chinese or English.

#### 2.4.2 Exclusion criteria

Studies were excluded if they met any of the following criteria: (1) animal studies, case reports, reviews, commentaries, or other non-original articles; (2) studies that did not focus on the effects of digital wearable devices on exercise self-management behaviors in patients with T2DM; (3) duplicate publications or studies with incomplete data; (4) studies for which the full text was unavailable; or (5) studies involving populations other than patients with T2DM.

### 2.5 Study selection

All retrieved records were imported into EndNote 20 for duplicate removal. Two reviewers, both trained in evidence-based research methods, independently screened the titles and abstracts according to the predefined inclusion and exclusion criteria. Full-text articles deemed potentially eligible were then retrieved and assessed for inclusion. Any disagreements arising during the screening process were resolved through discussion with a third reviewer until consensus was reached.

### 2.6 Data extraction

Data extraction included the following items: author information, year of publication, country of study, study design, sample size, participant characteristics, device type, intervention/application content, main physical activity-related outcomes, behavior change-related elements, and theoretical underpinnings. All extracted data were systematically organized using a standardized data extraction form to ensure consistency and facilitate subsequent analysis and tabular presentation. Because a formal risk-of-bias assessment was not planned for this scoping review, descriptive indicators of methodological strength were also extracted to contextualize the evidence. These indicators included study type, such as pilot, feasibility, or developmental studies; sample size; study design; intervention duration; comparator characteristics; device type; and outcome domain.

### 2.7 Data analysis

#### 2.7.1 Text preprocessing

Text mining was performed to identify high-frequency keywords and core concepts from the 11 included studies. After rigorous preprocessing, including the removal of 181 DOI/reference-related sentences and 332 author-list sentences, a final corpus of 2,487 valid sentences was obtained. The processed corpus contained 29,142 words and 6,886 unique vocabulary items, with a mean of 11.7 words per sentence.

#### 2.7.2 Topic modeling using LDA

Latent Dirichlet Allocation (LDA) was applied to identify latent thematic structures within the corpus. The optimal number of topics was determined by systematically comparing coherence scores and perplexity values across models with K ranging from 2 to 10.

#### 2.7.3 BERT-based semantic clustering

To complement the lexical-level topic modeling, BERT-based semantic clustering was conducted using the pre-trained bert-base-uncased model. A 768-dimensional embedding vector was generated for each of the 2,487 sentences. The optimal number of clusters was identified through joint evaluation of the sum of squared errors (SSE) and silhouette scores.

#### 2.7.4 Keyword co-occurrence network analysis

A domain-specific keyword co-occurrence network analysis was performed using a predefined keyword dictionary that covered eight conceptual categories: digital wearable devices, self-monitoring, real-time feedback, goal setting, motivation and adherence, exercise behavior, glycemic outcomes, and behavior change theory.

#### 2.7.5 Co-occurrence pattern analysis

Using a predefined keyword dictionary, sentence-level classification was performed for text related to digital wearable devices, exercise behavior, and behavior change pathways in the included studies. The co-occurrence frequencies of different conceptual categories within the same text unit were then calculated. Based on these results, a keyword co-occurrence network and the co-occurrence relationships between digital wearable devices and each category of behavior change pathways were constructed to identify the strength and structural characteristics of conceptual associations.

#### 2.7.6 Software and version control

Text mining and machine learning analyses were primarily conducted in the Python environment. Libraries including pandas, nltk, scikit-learn, gensim, and sentence-transformers were used for text preprocessing, topic modeling, semantic representation, and clustering analysis. To ensure reproducibility, the raw data, cleaned text, analysis scripts, and result files were systematically stored and managed under version control.

#### 2.7.7 Quality control and manual validation

To improve the reliability of the analytical results, irrelevant information and anomalous terms were manually reviewed and removed during the text preprocessing stage. After topic modeling and clustering analysis, two researchers reviewed the results in conjunction with the original study content and performed semantic refinement where necessary to ensure the accuracy of topic classification and pathway identification.

#### 2.7.8 Rationale for model selection

A combined analytical approach integrating LDA, BERT-based semantic representation, clustering, and co-occurrence analysis was adopted in this study. LDA and BERT were selected because they provide complementary perspectives on the textual corpus. LDA identifies recurring topics on the basis of word-distribution patterns and estimates the relative prominence of these topics across the corpus, whereas BERT-based semantic representation captures contextual similarities between passages that may use different terminology. Clustering was subsequently used to organize semantically related passages, and co-occurrence analysis was applied to quantify the relationships among digital wearable device functions, behavior change processes, and exercise-related outcomes. In contrast to conventional thematic synthesis, which depends primarily on reviewer-defined coding and study-by-study interpretation, the computational analyses examined the complete sentence-level corpus and quantified latent structures across studies. These analyses were not used to estimate intervention effects or establish causal relationships. Rather, they supplemented the manual synthesis by revealing corpus-wide patterns, underrepresented themes, and cross-study semantic relationships that might be difficult to identify consistently through narrative review alone. All algorithm-derived themes and clusters were independently reviewed by two researchers against the source articles before they were interpreted and incorporated into the final behavior change pathway framework.

## 3 Results

**Figure 1:**
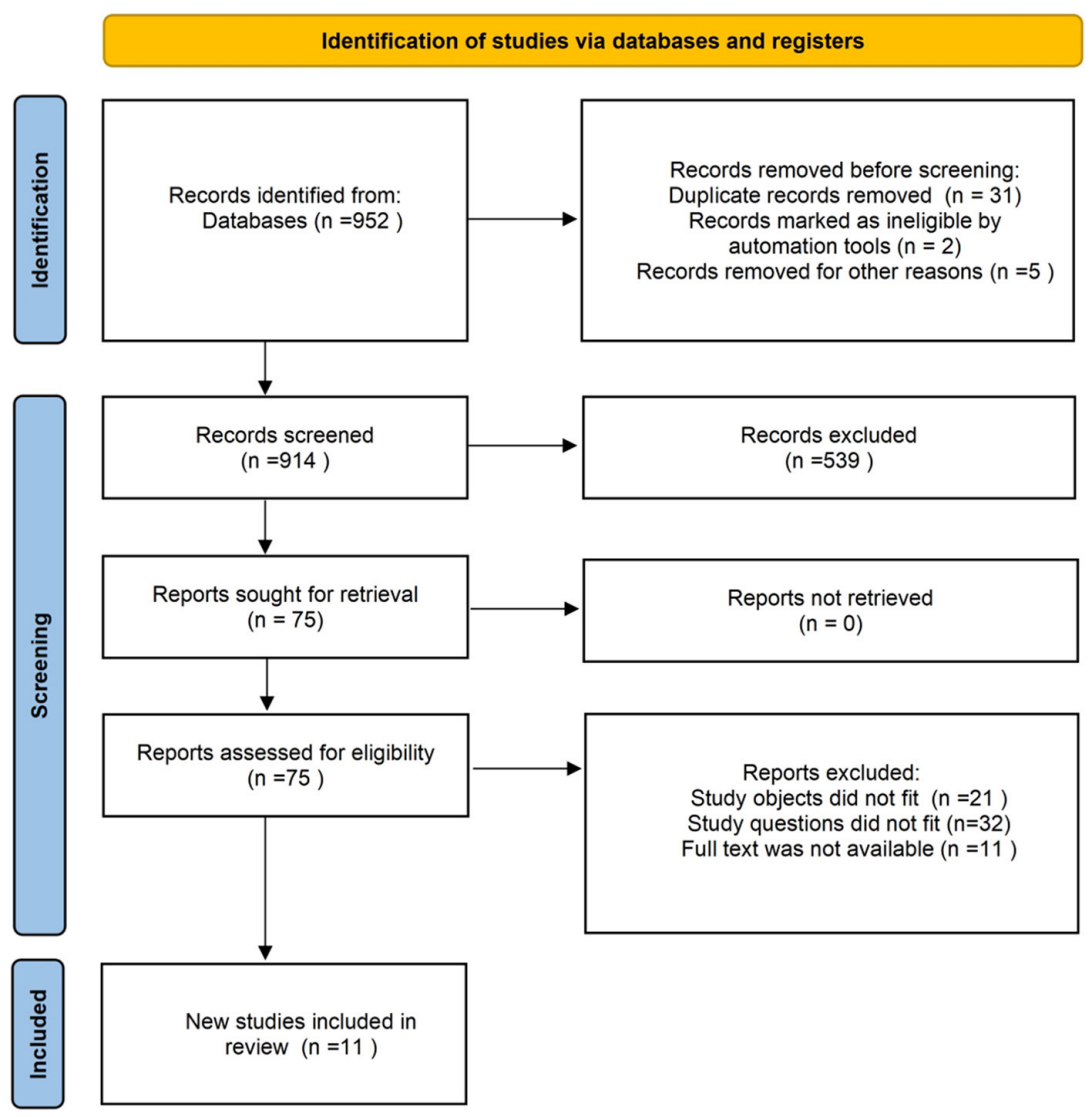
Literature selection flowchart illustrating the identification, screening, eligibility assessment, and inclusion stages in the scoping review on behavior change pathways by which digital wearable devices support exercise self-management in adults with type 2 diabetes mellitus (Scoping review, global, 2026).

A total of 952 records were identified through the initial database search. Following screening based on the predefined inclusion and exclusion criteria, 11 studies were ultimately included in this scoping review. The detailed study selection process is shown in Figure 1.

### 3.1 Characteristics of included studies

The 11 included studies were conducted in seven countries: the United States (*n* = 3), Canada (*n* = 2), Australia (*n* = 2), the Netherlands (*n* = 1), Japan (*n* = 1), Turkey (*n* = 1), and Spain (*n* = 1). Study designs included seven randomized controlled trials, one user-centered design study, one pilot study, one two-arm non-randomized controlled study, and one stratified pragmatic controlled trial. Detailed information regarding methodological characteristics, participant characteristics, device types, intervention content, main physical activity-related outcomes, behavior change-related elements, and theoretical basis is provided in Table 1.

**Table 1:** Key characteristics of studies on behavior change pathways by which digital wearable devices support exercise self-management in patients with type 2 diabetes (Scoping review, global, 2026).

References	Year of publication	Country	Study design	Sample size (*n*)	Participant characteristics	Device type	Intervention/ application content	Primary physical activity–related outcomes	Behavior change components	Theoretical foundation
Paschali A A, et al. (16)	2005	USA	RCT	26	Obese adults with Type 2 diabetes, sedentary, aged 30–65, BMI ≥30 kg/m^2^, diagnosed with Type 2 diabetes for at least 1 year	Triaxial accelerometer (BioTrainer, IM Systems)	Monthly behavioral counseling for 3 months; feedback group received accelerometer data feedback, nonfeedback group received counseling based on self-reports	Feedback group showed a trend toward increased activity, whereas the nonfeedback group returned toward baseline by the end of the study; however, the hypothesized between-group effect was not statistically significant.	Objective feedback, self-regulation of exercise intensity, goal-setting	Goal-setting and self-regulation-related rationale (the intervention was theory-informed rather than explicitly theory-based).
Allen N A, et al. (19)	2008	USA	RCT	52	Adults with Type 2 diabetes, not requiring insulin, inactive, HbA1c > 7.5%	CGMS + accelerometer	Both groups received 90 min of diabetes education and a follow-up call at week 4; the intervention group additionally received CGMS-based counseling derived from self-efficacy theory, including role-model glucose graphs illustrating the effect of physical activity on glucose levels	The intervention group showed improved physical activity self-efficacy, reduced sedentary/light activity, increased moderate activity, and reduced HbA1c and BMI	Self-efficacy enhancement, real-time physiological feedback, role modeling, behavioral counseling	Social Cognitive Theory/self-efficacy theory
Allen N, et al. (20)	2011	USA	RCT	29	Women with T2DM; aged 30–65 years; insufficiently active; HbA1c >7.0%	CGM+ ActiGraph accelerometer	The intervention group received CGM counseling plus problem-solving training; the comparison group received CGM counseling plus general diabetes education; intervention duration was 12 weeks	The intervention group showed significantly greater problem-solving skills; moderate activity, dietary adherence, and weight outcomes improved more favorably, although several differences were not statistically significant	Physiological feedback, problem-solving, behavioral counseling, self-efficacy support	Problem-solving-oriented intervention; the article indicated that problem-solving may enhance self-efficacy and diabetes self-management
Van der Weegen S, et al. (14)	2013	The Netherlands	UCD study	31	Adults with T2DM	Triaxial activity sensor + smartphone app + secure website	Development and refinement of an activity monitoring and feedback tool integrated into primary care; the app displayed activity levels, goal attainment, and historical records, while healthcare professionals could review data online and negotiate goals	This was primarily a development/usability study rather than an efficacy trial; the main outcomes concerned prototype development and usability/acceptability	Self-monitoring, goal setting, feedback, professional support	Fogg's persuasive technology model
Bailey K J, et al. (17)	2016	Canada	Pilot study	13	Adults with prediabetes or T2DM; aged 18–75 years	Real-time continuous glucose monitor	An 8-week group-based physical activity self-monitoring intervention using CGM to observe glucose responses to exercise, combined with goal setting and group discussion	Self-monitoring and goal-setting self-efficacy improved; attendance and re-enrollment rates were high; fitness, waist circumference, and quality of life improved	Self-monitoring, goal setting, self-efficacy, group support	Social Cognitive Theory + group-mediated cognitive behavioral approach
Pelletier C, et al. (21)	2021	Canada	Pilot RCT	30	Type 2 diabetes patients, PA < 150 min/week, aged 18–90, stable medical condition	Fitbit Charge HR activity tracker, iPad	3-month intervention with PA promotion, personalized by a kinesiologist; tracker use in intervention group	Physical activity increased in both groups, with a larger increase in the intervention group; acceptability was high, HDL improved, and HbA1c showed a favorable trend without a clear significant between-group effect.	Autonomous motivation, feedback from activity tracker, goal setting	Motivation-focused assessment; discussion informed by Self-Determination Theory rather than an explicitly theory-guided intervention.
Tanaka R, et al. (22)	2022	Japan	2-arm non-RCT	62	Adults with T2DM	Accelerometer with real-time intensity display	Both groups were advised to perform at least 150 min/week of moderate-to-vigorous physical activity; the intervention group additionally used real-time monitoring for 10 days at baseline and again after 3 months	No significant overall between-group differences were observed; subgroup analyses showed increased MVPA and step counts among initially inactive participants, while active participants maintained MVPA; HbA1c and BMI did not significantly change	Intensity self-monitoring, real-time visual feedback, self-management	Not explicitly stated
Timurtas E, et al. (15)	2022	Türkiye	Stratified PCT	84	Adults with T2DM	Mobile app, smartwatch, and supervised exercise delivery modes	A 12-week individualized exercise program comparing supervised exercise, mobile app-based exercise, and smartwatch-based exercise; training included aerobic, resistance, flexibility, balance, and coordination components	No significant between-group differences were found in HbA1c or 6-minute walk test outcomes; overall improvements were observed across groups	Remote support, self-management, technology-assisted exercise implementation	Not explicitly stated
Coombes J S, et al. (18)	2022	Australia	Pilot RCT	30	Adults with T2DM not meeting physical activity guidelines	Wrist-worn heart rate monitor + PAI mobile app	A 12-week PAI e-Health program including wearable monitoring, PAI score tracking, and exercise plus behavioral counseling sessions	The PAI group improved exercise capacity, sleep duration, and body fat measures; most participants intended to continue using the system	Self-monitoring, individualized feedback, behavioral counseling, reinforcement/ support	Not explicitly stated; the program mainly reflected self-monitoring, individualized feedback, and behavioral counseling support.
Alòs F, et al. (25)	2025	Spain	RCT	54	Office employees with T2DM; aged 18–65 years; smartphone users	Walk@Work-App + MetaWear thigh-worn sensor (intervention); ActivPAL monitor used for outcome assessment	A 13-week mHealth intervention promoting “sit less and move more,” with real-time app-based feedback on sitting, standing, and stepping, along with individualized goals and motivational prompts	At 12 months, HbA1c, systolic blood pressure, and diastolic blood pressure were reduced; sedentary breaks increased, time in short sedentary bouts increased, and some leisure-time sedentary behaviors decreased	Self-monitoring, real-time feedback, goal setting, motivational prompts, ongoing support	No specific classical behavioral theory was explicitly reported
Chang C R, et al. (26)	2025	Australia	RCT	42	Adults with T2DM; aged 40–70 years; HbA1c 6.5%−9.0%; BMI 27–40 kg/m^2^; insufficiently active	CGM	An 8-week CGM-guided exercise-timing intervention based on each participant's 14-day glycemic profile; participants were assigned to pre-peak exercise, post-peak exercise, or wait-list control	No significant intervention effect on HbA1c; both exercise groups showed reduced 24-hour peak glucose and improved endothelial function, with high adherence	Individualized feedback, precision exercise prescription, self-management	Not explicitly stated

CGM, continuous glucose monitoring system; RCT, randomized controlled trial; T2DM, type 2 diabetes mellitus; UCD study, user-centered design study; Pilot RCT, pilot randomized controlled trial; 2-arm non-RCT, two-arm non-randomized controlled study; MVPA, moderate-to-vigorous physical activity; BMI, body mass index; Stratified PCT, Stratified pragmatic controlled trial; PAI mobile app, personal activity intelligence mobile application; HbA1c, Hemoglobin A1c.

The methodological characteristics of the included evidence indicated that the field remains at a relatively early stage of development. Five of the 11 studies were explicitly described as pilot or preliminary studies, and one was a user-centered design study primarily focused on intervention development and usability rather than effectiveness. Sample sizes ranged from 13 to 84 participants, with a median sample size of 31. Considerable heterogeneity was also observed in device types, intervention components, intervention duration, comparator conditions, and outcome measures. Some studies used continuous glucose monitoring systems, whereas others used accelerometers, activity trackers, smartwatches, or multicomponent mobile health systems. Therefore, the findings should be interpreted as a descriptive mapping of an emerging and heterogeneous evidence base rather than as comparative estimates of intervention effectiveness.

### 3.2 Keyword distribution and co-occurrence patterns

**Figure 2:**
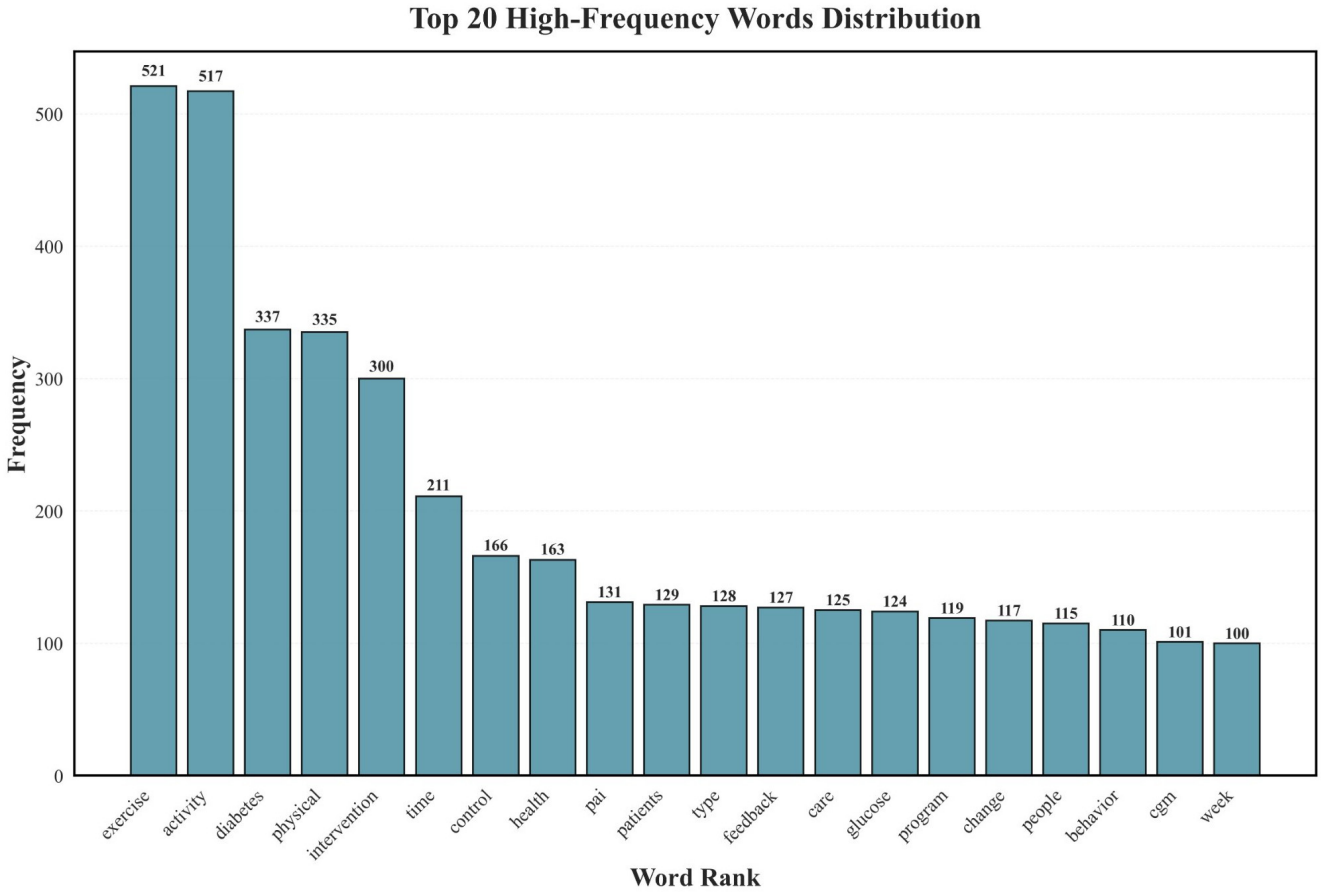
High-frequency keyword analysis depicting core concepts discussed in studies on behavior change pathways by which digital wearable devices support exercise self-management in adults with type 2 diabetes mellitus (Scoping review, global, 2026).

High-frequency keyword analysis showed that the most frequently occurring terms were exercise (521 occurrences, 1.79%), activity (517, 1.77%), diabetes (337, 1.16%), physical activity (335, 1.15%), and intervention (300, 1.03%). These findings indicate that the existing literature has consistently focused on diabetes management interventions centered on physical activity and exercise. Notably, feedback appeared 127 times (0.44%) and glucose monitoring appeared 124 times (0.43%), reflecting a dual emphasis on real-time biofeedback mechanisms and glycemic monitoring in the included studies. In addition, terms such as behavioral intervention (110, 0.38%) and continuous glucose monitoring (CGM; 101, 0.35%) further highlight the behavior-oriented nature of the field and the prominent role of CGM technology. Detailed results are presented in Figure 2.

### 3.3 Topic structure analysis

**Figure 3:**
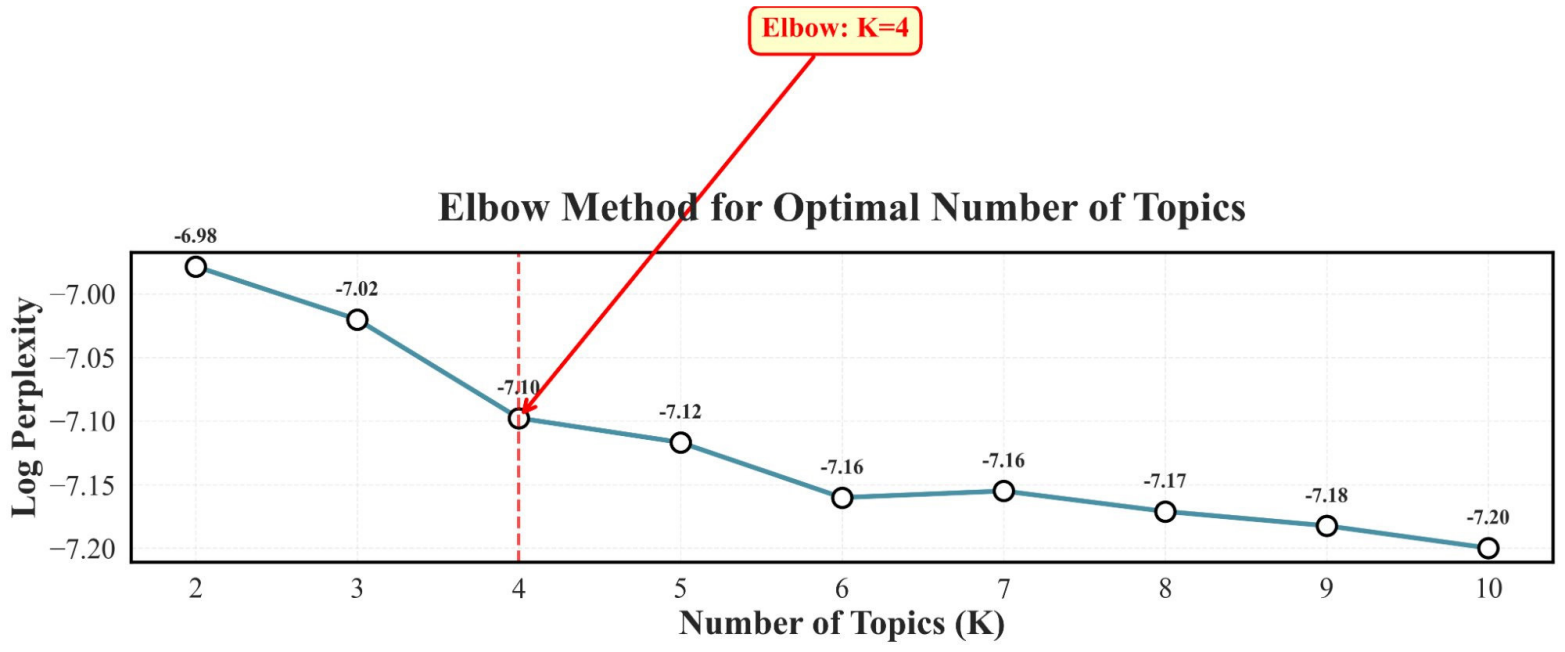
Elbow method plot of log-perplexity for selecting the optimal number of LDA topics in the scoping review on digital wearable devices supporting exercise self-management in adults with type 2 diabetes mellitus (Scoping review, global, 2026).

The topic structure analysis showed that, based on the elbow point identified from the log perplexity curve, *K* = 4 was selected as the inflection point, at which the rate of decrease in perplexity markedly slowed (from −7.10 at *K* = 4 to −7.12 at *K* = 5). The coherence score increased gradually from 0.3212 at *K* = 2 to a peak of 0.3879 at *K* = 9; however, beyond K = 4 (coherence = 0.3592), the marginal improvement was limited. Taking into account statistical parsimony and interpretability of the topic content, *K* = 4 was ultimately selected as the optimal number of topics (Figure 3).

**Figure 4:**
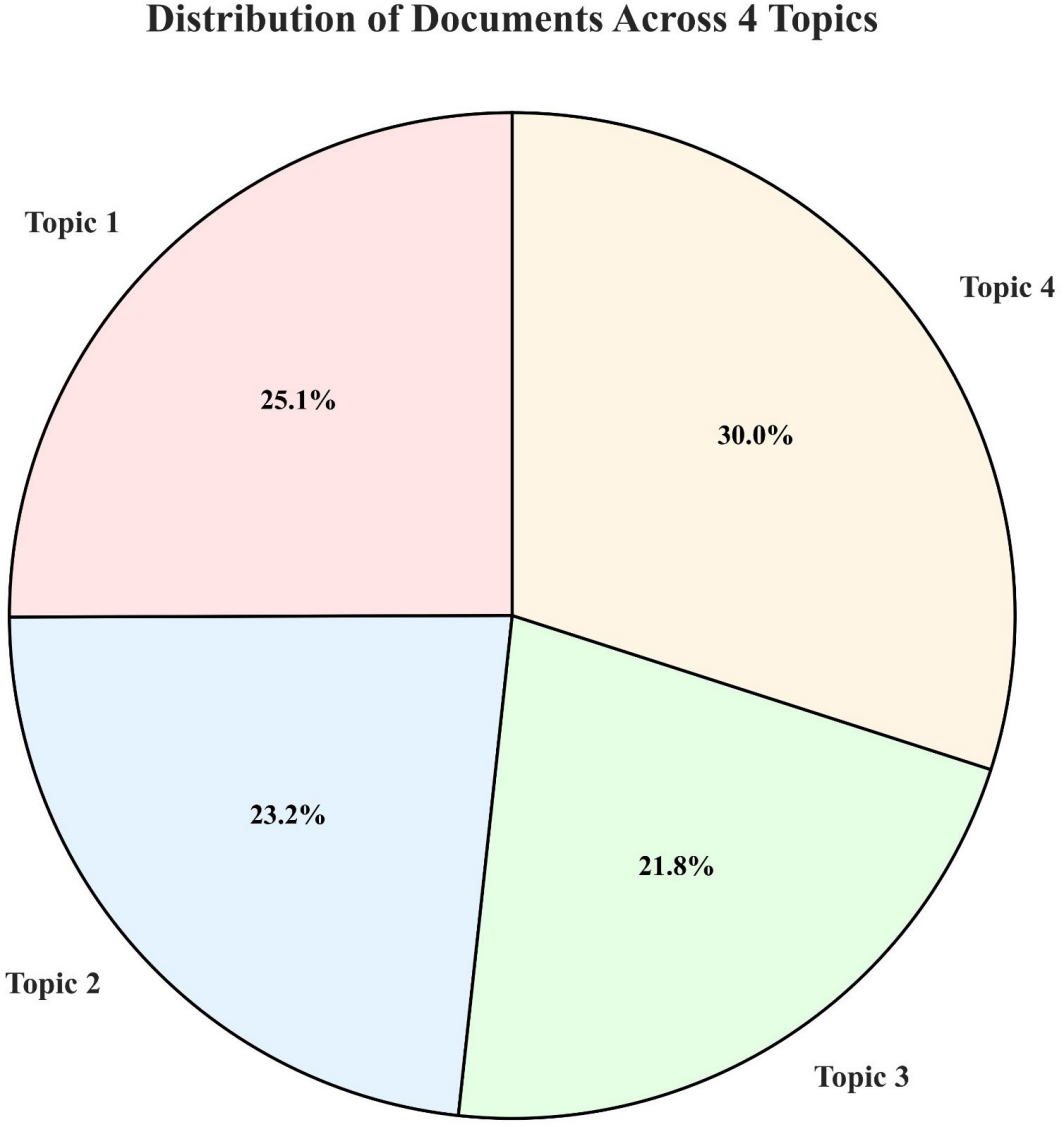
Proportional distribution of documents across four LDA topics in the scoping review on digital wearable devices supporting exercise self-management in adults with type 2 diabetes mellitus (Scoping review, global, 2026).

The distribution of the top 20 keywords across the four LDA topics further illustrated distinct thematic patterns. Topic 1 (25.1%) was centered on exercise, interventions, behavior change, trial design, and adherence, with exercise showing the highest individual word weight across all topics (0.0463). This topic primarily reflected intervention-oriented studies, particularly randomized controlled trials evaluating exercise adherence outcomes. Topic 2 (23.2%) included terms related to weeks, diabetes, behavioral regulation, motivational factors, and problem-solving capacity, representing a behavioral self-regulation dimension characterized by weekly monitoring cycles and motivational strategies. Topic 3 (21.8%) was characterized by intervention, glucose levels, diabetes management, time-related dimensions, and control mechanisms, reflecting a domain focused on glycemic monitoring and clinical outcome assessment. Within this topic, continuous glucose monitoring (CGM; 0.0137) and blood testing (0.0136) were notable indicators of clinical application value. Topic 4 (30.0%) focused on activity volume, exercise intensity, physical activity, time management, and activity trackers, with activity volume showing the highest keyword-pair weight across all topics (0.0545). This topic reflected research on physical activity tracking and digital wearable device applications, including references to specific devices such as Fitbit (0.0063) and the Personal Activity Intelligence (PAI) metric system (Figure 4).

### 3.4 BERT-based semantic clustering analysis

**Figure 5:**
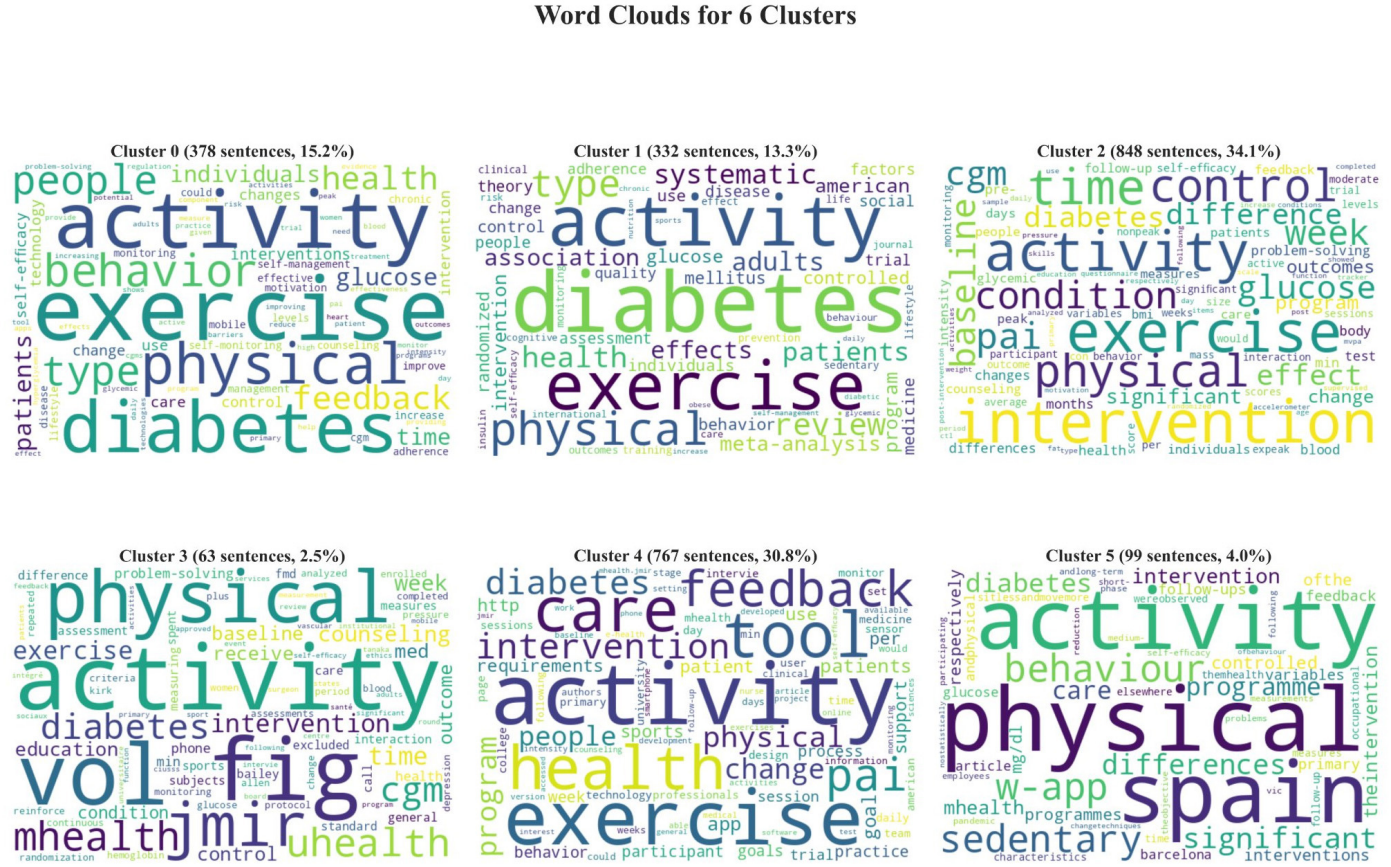
Word cloud visualization of six BERT semantic clusters in the scoping review on digital wearable devices supporting exercise self-management in adults with type 2 diabetes mellitus (Scoping review, global, 2026).

The BERT-based semantic clustering analysis divided the entire corpus into six semantic clusters. The clustering results indicated that research in this field mainly focused on the design and implementation of exercise interventions, device-based monitoring and feedback functions, goal setting and behavior maintenance, glycemic control-related outcomes, patient adherence and motivational support, and content related to behavior change theories. Compared with the LDA results, BERT-based clustering was better able to capture the deeper semantic relationships across different research themes, suggesting that exercise self-management supported by digital wearable devices is characterized by multidimensional interactions among technological, behavioral, and clinical components (Figure 5).

### 3.5 Keyword co-occurrence network analysis

**Figure 6:**
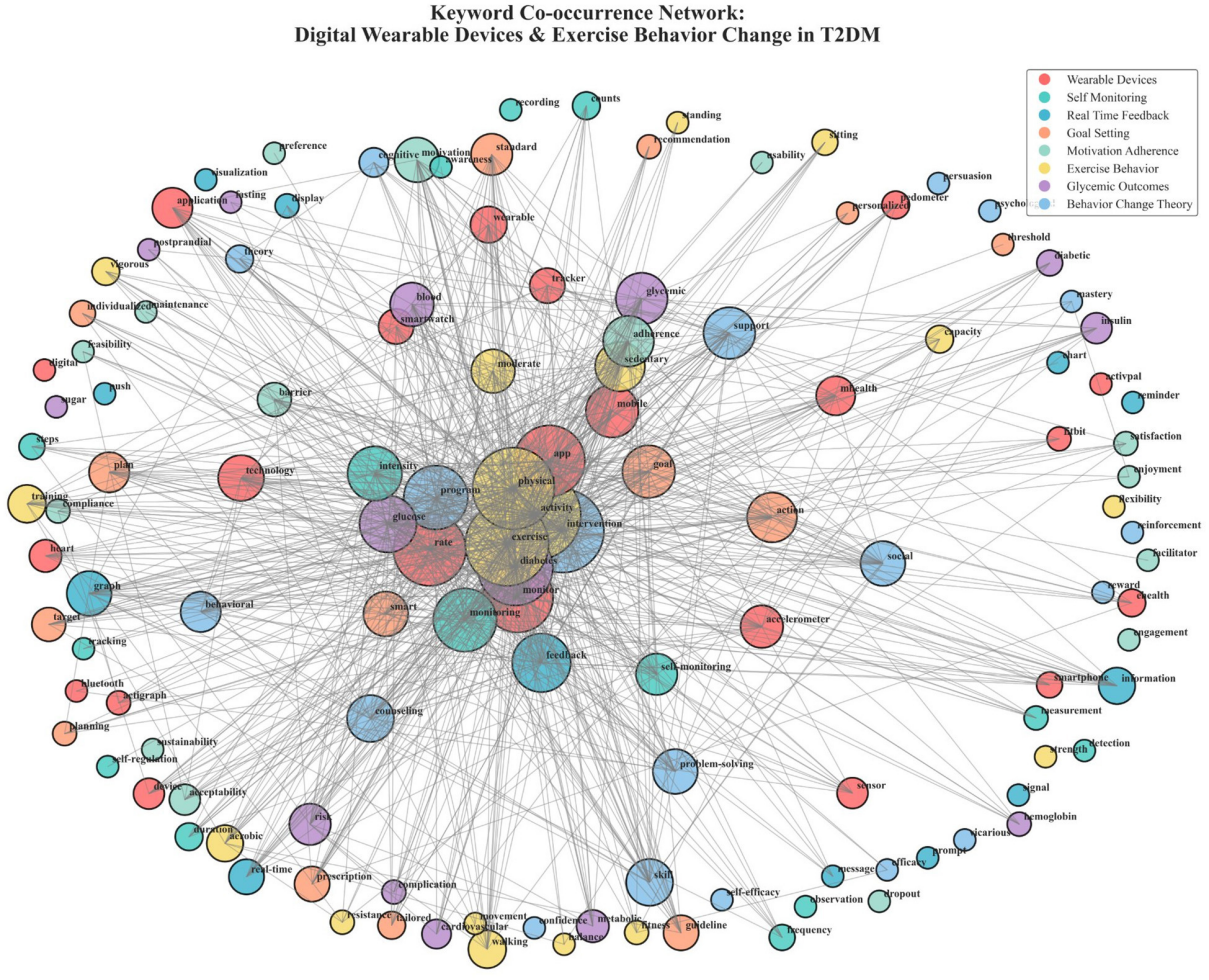
Keyword co-occurrence network depicting digital wearable devices and exercise behavior change in adults with type 2 diabetes mellitus (Scoping review, global, 2026).

The keyword co-occurrence network showed a clear modular organizational structure, within which several concept clusters could be identified. The core region of the network consisted of highly connected nodes such as exercise, activity, body, intervention, and diabetes. These nodes appeared to function as bridging concepts, linking the technological domain (e.g., monitoring devices, trackers, sensors, mobile applications, and feedback mechanisms) with the behavioral domain (e.g., goal setting, motivational drivers, adherence, self-monitoring, and counseling support), as well as the clinical outcome domain (e.g., glycemic indicators, blood glucose levels, blood-based biomarkers, insulin levels, and cardiovascular indicators). Nodes related to behavior change theory—including self-efficacy, cognitive mechanisms, behavioral patterns, mastery, and social factors—were located in the more peripheral regions of the network while maintaining connections with both technological and behavioral nodes. This pattern suggests that these constructs may play an intermediary role in the pathway linking digital wearable device functions to behavior change processes (Figure 6).

### 3.6 Co-occurrence pattern analysis

**Figure 7:**
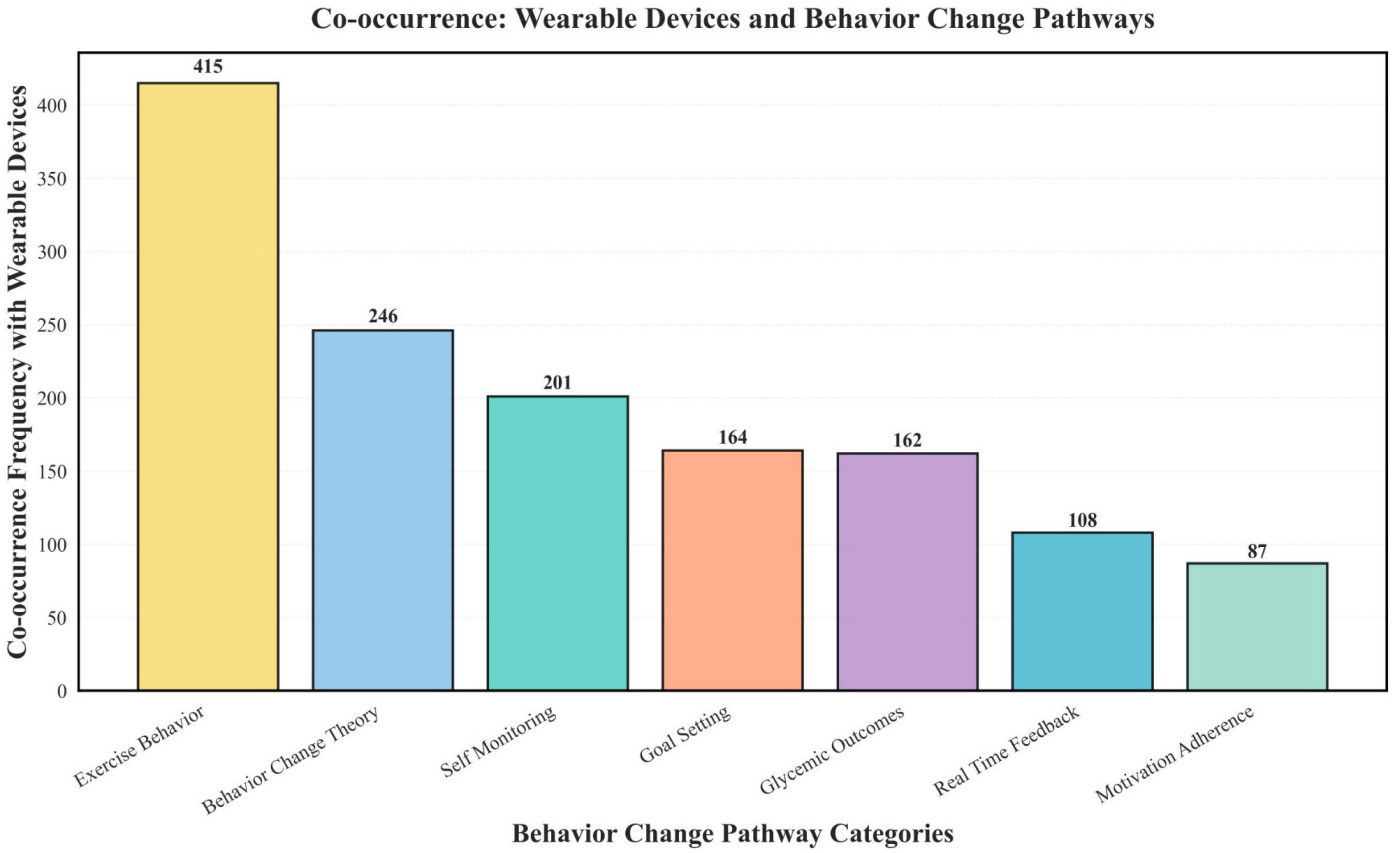
Co-occurrence frequency between digital wearable devices and behavior change pathway categories in adults with type 2 diabetes mellitus (Scoping review, global, 2026).

The co-occurrence relationships between digital wearable devices and each category of behavior change pathways demonstrated a clear gradient of association strength. Exercise behavior showed the highest co-occurrence frequency with digital wearable devices (415 occurrences), followed by behavior change theory (246), self-monitoring (201), goal setting (164), glycemic indicators (162), real-time feedback (108), and motivation/adherence (87). This gradient suggests that, although the association between digital wearable devices and exercise behavior itself was the most directly discussed in the literature, digital wearable devices were also consistently linked to foundational mechanisms such as self-monitoring and goal setting. The relatively lower co-occurrence frequency for motivation/adherence (87) indicates that the current literature may have paid comparatively less attention to how digital wearable device use influences motivational processes and adherence-related outcomes (Figure 7).

### 3.7 Extraction of behavior change pathways

**Figure 8:**
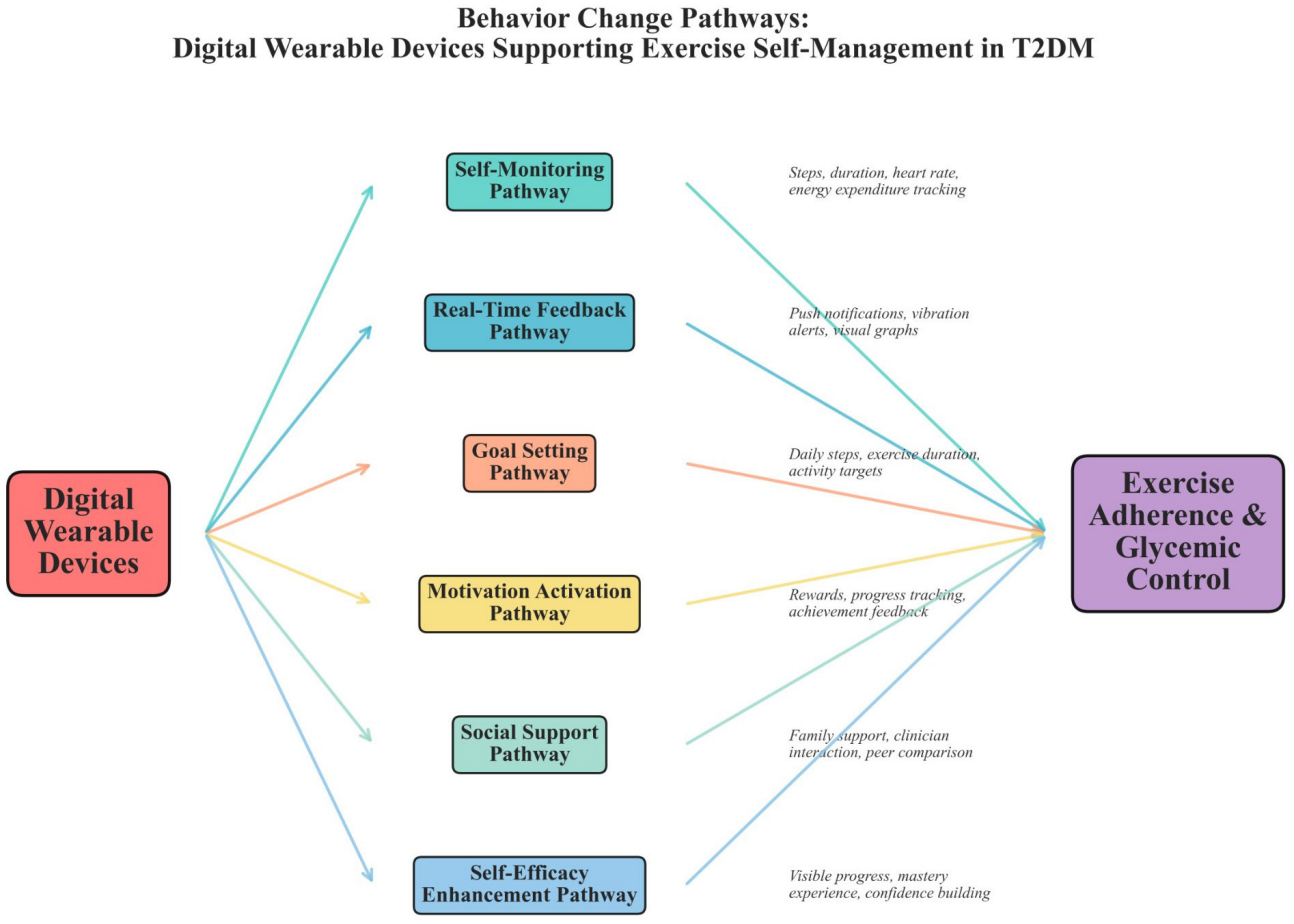
Schematic representation of behavior change pathways by which digital wearable devices support exercise self-management in adults with type 2 diabetes mellitus (Scoping review, global, 2026).

Based on the multidimensional evidence derived from topic modeling, semantic clustering, keyword network analysis, and co-occurrence pattern analysis, six core behavior change pathways were identified through which digital wearable devices may support exercise self-management in patients with T2DM: (1) a self-monitoring pathway, through which devices track step counts, activity duration, heart rate, and energy expenditure to enhance behavioral awareness; (2) a real-time feedback pathway, whereby push notifications, vibration reminders, and visualized data displays facilitate immediate behavioral adjustment; (3) a goal-setting pathway, in which daily step goals, exercise duration targets, and activity thresholds help establish a structured behavioral target system; (4) a motivation activation pathway, in which reward mechanisms, progress tracking, and achievement feedback help sustain intrinsic motivation; (5) a social support pathway, through which family support, clinician interaction, and peer comparison functions reinforce exercise maintenance; and (6) a self-efficacy enhancement pathway, in which visualized progress and mastery experiences strengthen patients' confidence in exercise management (Figure 8).

The six pathways were not generated solely from the frequency of individual terms or from a single machine learning model. Instead, they were derived through triangulation of LDA topics, BERT-based semantic clusters, keyword co-occurrence patterns, and manual examination of the original studies. The computational analyses identified recurring thematic and semantic structures across the corpus, whereas the final naming, conceptual interpretation, and theoretical classification of the pathways were completed by the research team. This combined procedure reduced reliance on any single algorithm and helped distinguish repeatedly documented behavior-support processes from isolated or study-specific observations.

## 4 Discussion

### 4.1 Behavior change pathways through which digital wearable devices support exercise self-management in patients with type 2 diabetes mellitus

This review identified several interrelated behavior change pathways through which digital wearable devices may support exercise self-management in patients with T2DM. Evidence derived from topic modeling, semantic clustering, keyword co-occurrence analysis, and the original content of the included studies suggests that digital wearable devices do not act solely through isolated technical functions. Instead, their value appears to lie in the combined operation of self-monitoring, real-time feedback, goal setting, motivation activation, social support, and self-efficacy enhancement. In this sense, digital wearable devices contribute not only by recording or prompting exercise, but also by embedding behavior regulation into everyday life and supporting the initiation, adjustment, and maintenance of exercise behaviors (14, 15). Self-monitoring appears to be a foundational mechanism within this framework. Compared with traditional approaches that rely on recall or paper-based records, digital wearable devices provide continuous and objective tracking of step counts, activity duration, heart rate, and energy expenditure, thereby converting exercise behavior into visible and quantifiable information (14, 16–18). Such data may strengthen patients' awareness of their behavioral status and provide a basis for behavioral adjustment (14, 17, 19, 20). Real-time feedback further enhances the intervention potential of digital wearable devices by transforming data into actionable information through reminders, notifications, trend displays, and progress summaries (15, 17, 21, 22). Goal setting also seems central, as devices can translate broad recommendations into specific and feasible targets, such as daily step goals or activity thresholds (14, 17, 21). In addition, motivation activation, social support, and self-efficacy enhancement appear to be important for long-term maintenance. Reward mechanisms, progress tracking, and achievement feedback may help sustain engagement (15, 21–24), while family involvement, peer comparison, clinician interaction, and remote feedback may broaden the social context of exercise behavior (14, 15, 19). Over time, repeated goal attainment and visible progress may strengthen patients' sense of mastery and self-efficacy, thereby facilitating a shift from externally prompted behavior to proactive self-management (14, 17, 20, 25, 26). Taken together, these pathways suggest that digital wearable devices may offer a more continuous, interactive, and individualized approach to exercise management in T2DM.

### 4.2 Associations between digital wearable device–related behavior change pathways and behavior change theories

The behavior change pathways identified in this review are broadly consistent with several established behavior change theories. Self-monitoring, real-time feedback, and goal setting align closely with Social Cognitive Theory and self-regulation perspectives, as digital wearable devices support a closed-loop process of self-observation, comparison with goals, and behavioral adjustment (14, 17, 20, 27). Goal-setting functions are also consistent with Goal-Setting Theory, because digital wearable devices translate broad recommendations into specific and actionable targets such as daily step goals or activity thresholds (28). Motivation activation and social support may be interpreted through Self-Determination Theory, which emphasizes the importance of autonomy, competence, and relatedness in sustaining behavior (29). Features such as individualized goals, progress visualization, reward feedback, family involvement, peer comparison, and clinician interaction may help address these psychological needs (14, 19, 20). In addition, the self-efficacy enhancement pathway is closely linked to the concept of self-efficacy within Social Cognitive Theory. By enabling repeated goal attainment and visible progress, digital wearable devices may help patients build confidence in their ability to manage exercise, which may in turn support the long-term maintenance of exercise behavior (30). Taken together, these findings suggest that the functional design of digital wearable devices is not only technologically relevant, but also theoretically congruent with the psychological processes that underpin sustained behavior change.

### 4.3 Added value of machine learning–assisted text mining and comparison with previous reviews

Previous reviews have generally examined the effects of wearable technologies on broad healthcare outcomes in chronic diseases, the clinical application of smartwatch technologies in diabetes, home-based digital diabetes management, or the effects of wearable technologies on physical activity and metabolic outcomes (5, 6, 24, 31). These reviews have provided valuable evidence regarding device feasibility, acceptability, physical activity outcomes, and metabolic indicators. However, they have placed comparatively less emphasis on how specific digital wearable device functions may activate or support behavior change processes related to exercise self-management in people with T2DM. The present review therefore extends the existing literature by focusing specifically on the pathways linking device functions, such as self-monitoring and real-time feedback, to behavioral processes, including goal setting, motivation, social support, and self-efficacy. The machine learning–assisted component also provided information beyond that obtained through conventional narrative synthesis. LDA quantified the relative prominence of four major thematic domains, showing that physical activity tracking and digital wearable device applications constituted the largest topic, whereas behavioral self-regulation and motivation represented a smaller proportion of the literature. BERT-based clustering identified semantic relationships among passages that were expressed using different terminology, thereby linking technological, behavioral, and clinical content across studies. Co-occurrence analysis further demonstrated that motivation and adherence were discussed less frequently in association with digital wearable devices than exercise behavior, self-monitoring, or goal setting. These findings identify an imbalance in the existing literature and suggest that future studies should examine motivational maintenance and long-term adherence more explicitly. Nevertheless, the computational findings should not be interpreted as independent evidence of effectiveness or causality. Topic weights, cluster membership, and co-occurrence frequencies reflect patterns of discussion within the included literature rather than the magnitude of clinical effects. The principal value of the machine learning analyses was therefore to enhance the transparency, consistency, and breadth of evidence mapping, while the interpretation of the identified pathways remained grounded in manual examination of the included studies and relevant behavior change theories.

### 4.4 Implications for clinical practice and future research

From a clinical perspective, digital wearable devices may be incorporated into continuous care, remote follow-up, and individualized health management for patients with T2DM. Drawing on the six core behavior change pathways identified in this review, healthcare professionals may develop more targeted intervention strategies (32–34)^.^ For patients who have not yet initiated exercise behavior, greater emphasis may be placed on the self-monitoring and goal-setting functions of digital wearable devices to help build behavioral awareness, clarify behavioral targets, and facilitate behavior initiation (35). For patients who experience difficulty maintaining exercise behavior, greater attention may be directed toward the real-time feedback, motivation activation, and social support functions of these devices in order to provide sustained positive reinforcement and social support (36). Across intervention stages, a central focus should be placed on strengthening exercise self-efficacy. Through visualized progress trajectories and stage-based summaries of achievement, digital wearable devices may help patients accumulate successful experiences and gradually internalize exercise behavior over time (37). Future research should further strengthen the systematic application of behavior change theories in device development and intervention design. Greater attention is also warranted to individual differences across patient populations, long-term adherence, and the sustainability of intervention effects in real-world settings. In addition, the issue of the digital divide deserves careful consideration. Future studies should explore the applicability and acceptability of digital wearable devices among older adults and among patients with T2DM who have lower educational attainment or lower income, so as to provide a stronger evidence base for their standardized and equitable implementation (31, 38–41).

### 4.5 Limitations

This review has several limitations. First, only 11 studies met the eligibility criteria, and a substantial proportion of the evidence consisted of pilot, preliminary, feasibility, or developmental studies with relatively small sample sizes. The limited number and early-stage nature of the included studies reduce the certainty and generalizability of the identified patterns. In addition, considerable heterogeneity existed across digital wearable device types, intervention components, intervention duration, comparator conditions, theoretical foundations, and outcome measures, which limited direct comparison across studies. Second, consistent with the purpose of a scoping review, no formal risk-of-bias or methodological quality assessment was conducted. Although descriptive indicators of methodological strength were extracted to contextualize the evidence, the frequency with which a pathway appeared in the literature should not be interpreted as evidence that the pathway is effective. The mixture of randomized, non-randomized, pilot, and developmental designs also means that causal relationships between digital wearable device functions, behavior change processes, and exercise-related outcomes cannot be established. Because of the small and heterogeneous evidence base, publication bias could not be formally assessed. Furthermore, restricting inclusion to studies published in English or Chinese may have resulted in language-related selection bias. Third, the machine learning–assisted analyses were affected by the size and composition of the corpus, text preprocessing decisions, keyword dictionary construction, model selection, and parameter settings. LDA relies primarily on patterns of word distribution and may not fully capture contextual meaning, whereas BERT-based clustering may be influenced by the characteristics of the pretrained language model and the selected clustering procedure. Co-occurrence frequencies indicate that concepts appeared within the same textual units but do not establish the direction, strength, or causal nature of their relationships. Although manual validation by two researchers was used to improve interpretability, some degree of researcher judgment remained unavoidable in topic labeling and pathway classification.

## 5 Conclusion

In conclusion, the available evidence suggests that digital wearable devices may support exercise self-management in people with T2DM through several interrelated behavior change pathways, including self-monitoring, real-time feedback, goal setting, motivation activation, social support, and self-efficacy enhancement. Machine learning–assisted text mining helped identify and quantify recurring thematic and semantic patterns across the literature, but these patterns should not be interpreted as evidence of causal mechanisms or comparative intervention effectiveness. Given the small number of included studies, generally modest sample sizes, and substantial heterogeneity in devices and intervention designs, the findings should be regarded as a preliminary conceptual mapping of an emerging evidence base. Larger, theory-informed, and methodologically rigorous studies with longer follow-up are needed to determine which device functions and behavior change pathways most effectively support sustained exercise self-management in people with T2DM.

## Data Availability

The original contributions presented in the study are included in the article/supplementary material, further inquiries can be directed to the corresponding author.
